# The long road to TRAIL therapy: a TRAILshort detour

**DOI:** 10.18632/oncotarget.27902

**Published:** 2021-03-30

**Authors:** Aswath P. Chandrasekar, Mark Maynes, Andrew D. Badley

**Keywords:** TRAIL, TRAILshort, cancer therapy, apoptosis, anti-apoptosis

The goal of modern medicine is to cure disease and alleviate human suffering. In this endeavor, research has progressed at tremendous pace, allowing for revolutionary therapies that have improved the human condition and provided cures for diseases previously thought to be incurable. Our knowledge of the immunological processes necessary for the maintenance of health has never been more informed and immunotherapy has become a mainstay of therapy for a variety of health conditions.

**Figure 1:**
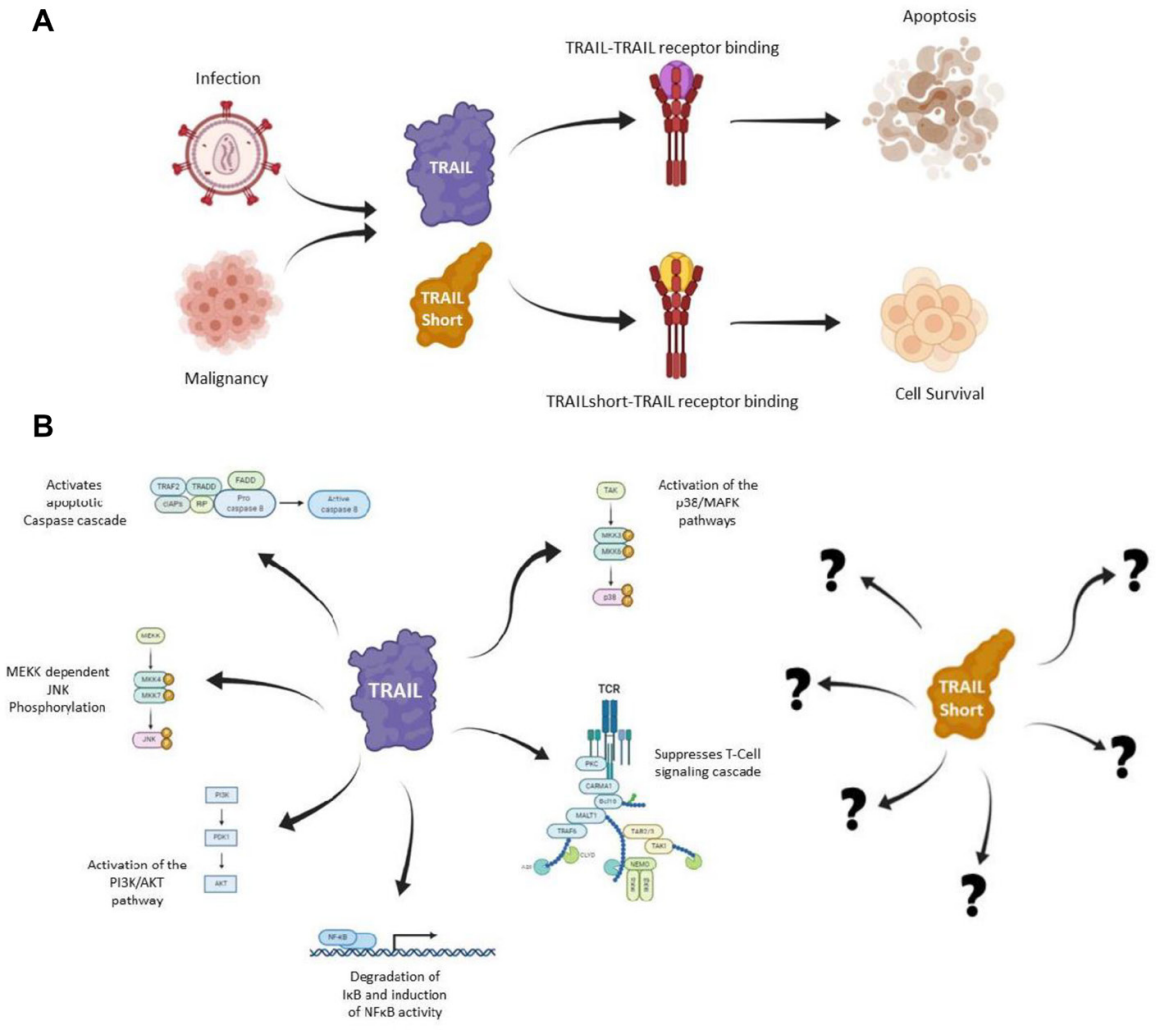
The functions of TRAIL and TRAILshort. (**A**) TRAIL and TRAILshort may both bind to the TRAIL receptors DR4 and DR5; however TRAILshort does not influence apoptosis. (**B**) Full length TRAIL has been demonstrated to influence a myriad of cell signaling cascades such as (clockwise from top): Caspase dependent apoptosis, activating the p38/MAPK pathway, suppressing TCR signaling and T-Cell proliferation, induction of NFκB activity, Activation of the PI3K/AKT pathway, and inducing MEKK dependent JNK phosphorylation, however the downstream effects of TRAILshort largely remain a mystery [28].

Across almost all infectious and malignant disease states, recovery requires a robust and targeted immune response. Immune effector elimination of target cells may be achieved by a variety of mechanisms such as the Perforin/Granzyme pathway or through ligands such as Fas Ligand, TNFα and the Tumor Necrosis Factor Related Apoptosis Inducing Ligand (TRAIL) [1–14]. These molecules function through their interaction with cognate receptors expressed on the surface of their targets and subsequently trigger a signaling cascade of intracellular cysteine-aspartic proteases (Caspases) that culminate in the cellular disintegration of their targets [15] (Figure 1A).

Of these death-inducing ligands, TRAIL has shown promise as a potential therapy. In the setting of cancers and chronic infections such as HIV, TRAIL has become recognized as an important immune effector molecule and an exciting avenue for therapeutic opportunity [3–5, 10]. This ligand functions mainly through its interactions with the surface receptors DR4 and DR5 to elicit apoptosis in diseased cells [16, 17]. Studies involving TRAIL for cancer therapy, while promising in *in-vitro* settings, were unfortunately disappointing in clinical trials [18].

In the setting of HIV infection, acutely infected cells were seen to be resistant to paracrine TRAIL-mediated apoptosis, an investigation into which lead to the discovery a dominant negative splice variant of TRAIL known as TRAILshort. This 11kDA, 101-amino acid protein was first identified in HIV-infected cells and is a splice variant of full-length TRAIL, containing exons 1, 2, and 5, with exons 3 and 4 absent. TRAILshort’s exclusion of cysteine 230 prohibits its trimerization; therefore, disrupting TRAIL inducing death [19]. This protein was seen to occupy the TRAIL receptors DR4 and DR5 without inducing cell cytotoxicity and prevent TRAIL-induced cell cytotoxicity. Further investigation revealed that antagonizing this protein with a monoclonal antibody resulted in the re-sensitization of TRAIL-resistant cells to TRAIL-mediated apoptosis [20].

In consideration of recent developments that identified that TRAILshort is present in CD4 cells from HIV naïve patients [21], the specificity of TRAILshort to HIV infection came into question. TRAILshort transcripts were found to be highly overexpressed in infectious diseases and upregulated in 40% of human malignancies, according to the GEO and TCGA databases. The presence of TRAILshort transcripts were associated with a total of 66 genes, of which 63 were Type I IFN regulated. This finding suggests that the presence of TRAILshort may be a type 1 interferon driven response, caused as a result of chronic immune stimulation. In fact, it has previously been demonstrated that TLR-7, TLR-8, TLR-9, and IFNα14 upregulated TRAILshort mRNA production in PBMCs [21]. In CD4 T cells, IFNα14 was seen to increase TRAILshort mRNA production as well [21].

The presence of TRAILshort in human malignant tissue was confirmed by both RNA ISH and immunohistochemistry (IHC), and while TRAILshort message was confined to malignant cells, TRAILshort protein was detected diffusely in the microenvironment, indicating a much wider affected footprint than the primary malignant tissue. The antagonism of TRAILshort by a targeted antibody was found to improve the ability of immune effector CD8 T Cells in clearing tumor cells in contrast to the effectors alone. Additionally, it was demonstrated that the presence of TRAILshort antibody significantly reduced tumor burden in mice that received treatment compared to those that did not.

The combination of these findings indicates a complex but significant role played by TRAILshort in the pathogenesis of disease. It remains to be elucidated if all the effects of TRAILshort are simply an antagonism of TRAIL-TRAIL receptor binding through competition for the cognate receptor or if TRAILshort exerts independent deleterious effects on immune effector cells through independent signaling mechanisms.

Full length TRAIL has been described as possessing several non-apoptotic functions across various cell types, mediated through the TRAIL: TRAIL-receptor axis. TRAIL has been shown suppress T Cell activation and cell proliferation by affecting T cell receptor signaling [22], increase the secretion of pro-inflammatory cytokines in an NFκB-dependent manner [23, 24], promote fibroblast expansion in rheumatoid arthritis [25], stimulate the growth of vascular smooth muscle endothelial cells [26], affect apoptotic signaling through a MEKK1 dependent pathway [27], and cause signaling in the p38 MAPK, JNK and AKT pathways [23, 27] (Figure 1B).

This contrasting myriad of both pro- and anti-inflammatory functions indicate the scope of the issue posed by TRAIL signaling in disease states. The contrasting effects thus far observed seem to be indicative that very selective micro-environmental and cell phenotypic conditions modulate the downstream effects of TRAIL signaling. This recent study therefore provides a platform for the expansion of TRAIL research in new directions and details a novel protein that may, in-part, precipitate the progression of disease and antagonize the mechanisms that serve to control it. Evidence thus far has revealed the increased expression of TRAILshort in cancerous cells and infected cells. Further studies are required to determine which cells specifically produce TRAILshort in a PBMC population, which may allow for better understanding of TRAILshort biology. Additionally, the observations thus far suggest that TRAILshort may be involved in protecting diseased cells from TRAIL mediated cell apoptosis facilitating their survival, however, given the myriad of signaling cascades that TRAIL has been described to induce, it stands to be determined if TRAILshort may induce similar phenotypes, affect T cell function, promote disease progression, and prove a hindrance to immune mediated therapeutic efforts.
